# Comparative effectiveness of transvaginal repair vs. hysteroscopic resection in patients with symptomatic uterine niche

**DOI:** 10.3389/fsurg.2023.1019237

**Published:** 2023-02-09

**Authors:** Wei Xia, Xiaofeng Wang, Yang Wang, Yuan Tian, Chuqing He, Chenfeng Zhu, Qian Zhu, Hefeng Huang, Liye Shi, Jian Zhang

**Affiliations:** ^1^ Department of Gynecology and Obstetrics, International Peace Maternity and Child Health Hospital, Shanghai Jiao Tong University School of Medicine, Shanghai, China; ^2^ Shanghai Key Laboratory of Embryo Original Diseases, Shanghai, China; ^3^ Obstetrics and Gynecology Hospital, Institute of Reproduction and Development, Fudan University, Shanghai, China; ^4^ Department of Ultrasound, International Peace Maternity and Child Health Hospital, Shanghai Jiao Tong University School of Medicine, Shanghai, China

**Keywords:** niches, cesarean section, hysteroscopic resection, transvaginal repair, postmenstrual spotting

## Abstract

**Objective:**

To compare the efficacy of transvaginal repair and hysteroscopic resection in improving niche associated postmenstrual spotting.

**Methods:**

The improvement rate of postmenstrual spotting in women who underwent transvaginal repair or hysteroscopic resection treatment was assessed retrospectively in patients accepted at the Niche Sub-Specialty Clinic in International Peace Maternity and Child Health Hospital between June 2017 and June 2019. Postoperative spotting symptom within one year after surgery, pre- and postoperative anatomical indicators, women' satisfaction with menstruation and other perioperative parameters were compared between the two groups.

**Results:**

68 patients in the transvaginal group and 70 patients in the hysteroscopic group were included for analysis. The improvement rate of postmenstrual spotting in the transvaginal group at the 3rd, 6th, 9th, and 12th months after surgery was 87%, 88%, 84%, and 85%, significantly higher than 61%, 68%, 66%, and 68% in the hysteroscopic group, respectively (*P* < 0.05). The total days of spotting improved significantly at the 3rd month after surgery but did not change over time within one year in each group (*P* > 0.05). After surgery, the disappearance rates of the niche are 68% in transvaginal group and 38% in hysteroscopic group, however, hysteroscopic resection had shorter operative time and hospitalization duration, less complications, and lower hospitalization costs.

**Conclusion:**

Both treatments can improve the spotting symptom and anatomical structures of uterine lower segments with niches. Transvaginal repair is better in thickening the residual myometrium than hysteroscopic resection, however, hysteroscopic resection has shorter operative time and hospitalization duration, less complications, and lower hospitalization costs.

## 1. Introduction

The increasing rate of cesarean section (CS) is a global public health challenge, accounting worldwide for 21.1% of all deliveries (1). Despite implementing various intervention strategies to reduce the CS rates in China, the overall rate has increased to nearly 40% (2, 3).The significance of the niche in the CS scar and their potential long-term complications in women's health have been previously recognized. The niche is reported in 49.6%∼64.5% of patients with at least one previous cesarean delivery (4–6). The prevalence increases with the number of cesarean deliveries, and it can even be as high as 100% especially with a history of three CSs (7). In addition to the number of CSs, several other variables may play a role in the development of this condition, such as maternal body mass index, gestational diabetes (8), and synthetic absorbable multifilament sutures for uterine closure (9).

The most common symptom of the niche that bothers women is postmenstrual spotting. Some reports described postmenstrual spotting in 29%–82% of patients with niche (5, 10), mainly manifesting as spotting at the end of menstruation, intermenstrual spotting, contact bleeding and heavy menstrual bleeding, which may severely affect women's quality of life. The niche also increases the risk of some late consequences during subsequent pregnancy, such as placenta praevia, placenta accreta spectrum disorders (11, 12). Clinically their expression in the first trimester is represented by the caesarean scar pregnancy (CSP) (13). In CSP, the gestation is implanted in a previous cesarean scar (14), however, it may lead life-threatening results, such as severe hemorrhage and uterine rupture. Although, management options have been described in case reports and small series in the published work (13, 15, 16), there is insufficient evidence to recommend any one specific intervention over another for CSP (17).

To date, guidelines for niche treatment are lacking, and the clinical treatment of niche-related spotting includes drug therapy and surgical treatments. Drug therapy can improve the postmenstrual spotting symptom of the niche but with obvious limitations, including the inability to restore the anatomy of the uterus, high recurrence rate after drug withdrawal, and significant side effects from long-term therapy. Surgical treatment is usually regarded as the optional treatment of the niche, including repair and resection. Repair surgery includes transvaginal repair, laparoscopic repair, and transabdominal repair surgery. Among them, transvaginal repair is the least invasive method with no scarring of the abdomen after surgery owing to the use of a natural orifice. Furthermore, transvaginal repair provides direct access to the cavity of the niche, complete resection of tissues surrounding the niche and full-thickness closure, improving the niche-related symptoms and quality of life (18–21). Hysteroscopic niche correction helps improve postmenstrual spotting and restore fertility by treating polyps and the lateral branches of the niche (ectopic endometrium and hyperplastic vessels) and removing their valves (outflow tract or inflow tract to allow the blood to flow smoothly) (22–24). Consequently, in recent years, transvaginal and hysteroscopic procedures have become the two most commonly used treatments of the niche with short operation time, fast postoperative recovery, and effective improvement of niche-related symptoms (15, 20, 21, 25, 26).

Treatments of the niche worldwide, regardless of the surgical procedures used, are poorly reported, and the post-operative outcomes remains unsatisfactory. The latest systematic review (27) included 33 publications, 28 of which investigated a single surgical technique and only five used comparative techniques; the meta-analysis showed that the effective rate of hysteroscopy in the improvement of bleeding symptoms was 85.00% (75.05–92.76%), while that of transvaginal repair was 82.52% (67.53–93.57%). It was also proposed that hysteroscopy had a lower risk of surgical complications. However, no studies have directly compared the efficacy of the transvaginal and hysteroscopic techniques in improving niche-related spotting to suggest which technique should be selected.

Therefore, here we compared the efficacy of transvaginal repair and hysteroscopic resection in improving niche-related postmenstrual spotting symptoms based on different observation time points after surgery and anatomical indicators.

## 2. Material and methods

### 2.1. Participants

In this retrospective cohort study, all women with niche who were diagnosed and treated at the Niche Sub-Specialty Clinic in the International Peace Maternity and Child Health Hospital (IPMCH) affiliated to Shanghai Jiao Tong University School of Medicine between June 2017 and June 2019 were included. Only women with the surgical history of transvaginal niche repair or hysteroscopic niche resection were included for analysis.

The inclusion criteria were as follows: (1) history of at least one CS; (2) existence of the symptom of postmenstrual spotting, defined as the presence of brown discharges for ≥2 days immediately after menstruation or intermenstrual bleeding for ≥2 days; and (3) the defect in the lower anterior uterine segment with at least a 2-mm depth by transvaginal ultrasonography (TVUS) and the residual myometrium thickness (RMT) between at least 2.2 mm. The exclusion criteria were as follows: (1) presence of abnormal menstruation before CS; (2) history of placement of a levonorgestrel intrauterine system or abnormal blood coagulation function or endocrine disease; (3) long-term use of oral contraceptives or gonadotropin-releasing hormone agonists; (4) presence of postoperative pathology-confirmed endometrial disease, such as submucosal fibroids, endometrial cancer, and abnormal endometrial hyperplasia; and (5) non-attendance for the routine outpatient visits.

In our sub-specialty clinic, all the women with niche who have received surgical or medical managements were followed by routine outpatient visits every three months for at least one year.

### 2.2. Evaluation criteria

The diagnosis of niche was based on medical history, clinical symptoms, and TVUS findings. Assessment of the spotting symptoms in our institute included self-reported days of spotting, visual analog scale (VAS) score for discomfort experienced with spotting, and Likert scale score (28) for menstrual satisfaction. Self-reported days of spotting were calculated as follows: total days of spotting = sum of the number of days of spotting at the end of menstruation and number of days of intermenstrual spotting. The VAS stratifies the symptom discomfort in a 0–10 scale: 0 points indicate “no discomfort,” and 10 points indicate “the most uncomfortable imaginable.” The Likert scale stratifies the patients based on satisfaction with menstruation in five grades: (1) strongly dissatisfied, (2) dissatisfied, (3) neither satisfied nor opposed, (4) satisfied, and (5) very satisfied.

**Figure 1:**
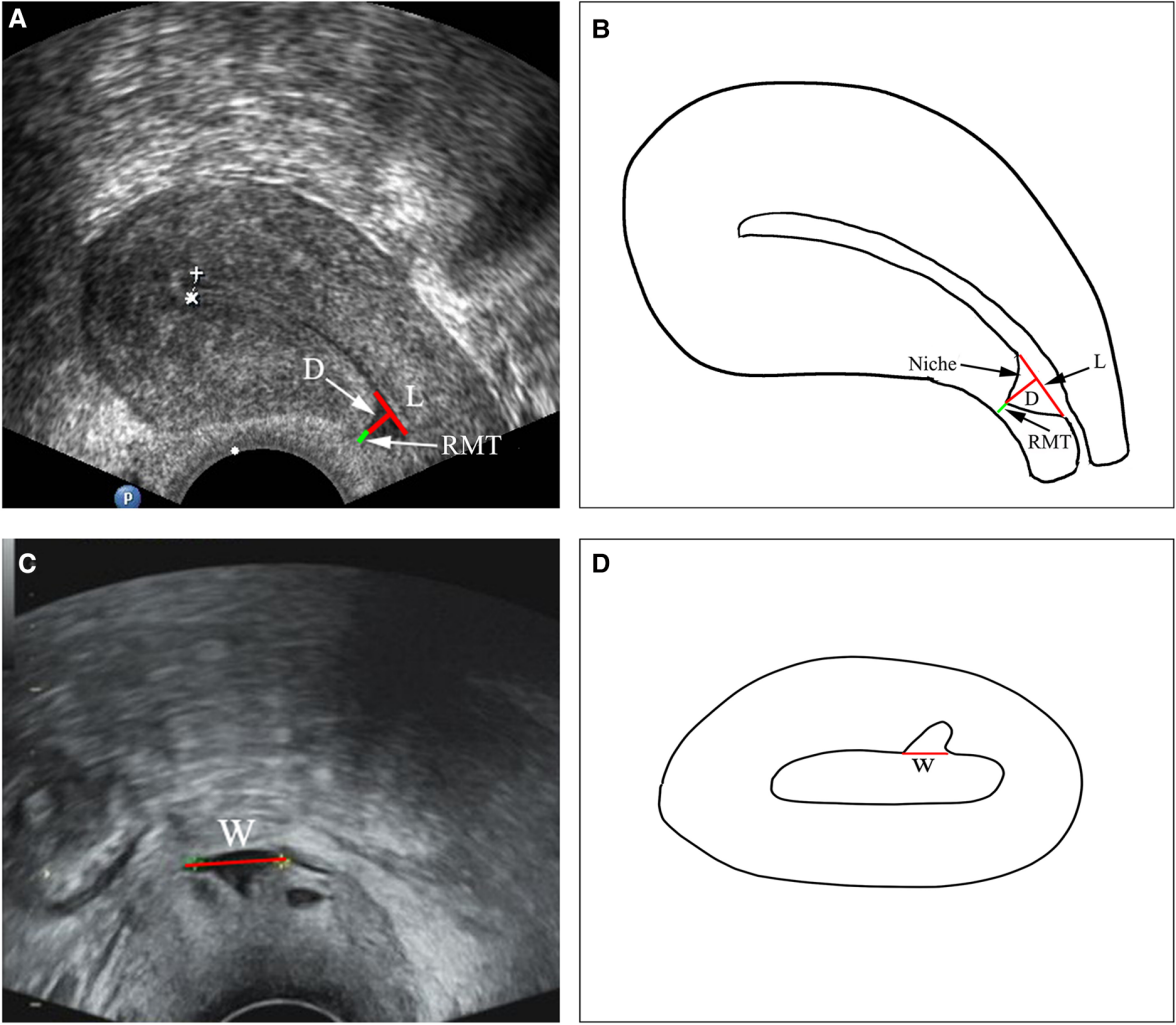
Ultrasonographic images of a uterine niche and schematic diagram demonstrating measurement of the niche. W, width; L, length; D, depth; RMT, residual myometrium thickness.

Before surgery, all patients underwent TVUS to assess the size of the niche and the relationship with the surrounding organs according to a standardized protocol (29). The parameters of the anatomical niche indicators were as follows: length (L), width (W), depth (D), and RMT, as shown in Figure 1.

### 2.3. Surgical procedures

To avoid the injury of bladder, all patients were evaluated by imaging before surgery and received surgery under general anesthesia by skilled gynecologists who had performed more than 300 transvaginal repair and hysteroscopic resection. Before surgery, patient's bladder was emptied. After surgery, 150 ml of methylene blue diluent filled the bladder to assess bladder integrity. In hysteroscopic surgery group, RMT was greater than 2.5 mm in most patients, with only seven patients' whose RMT ranging from 2.2 to 2.5 mm.

**Figure 2:**
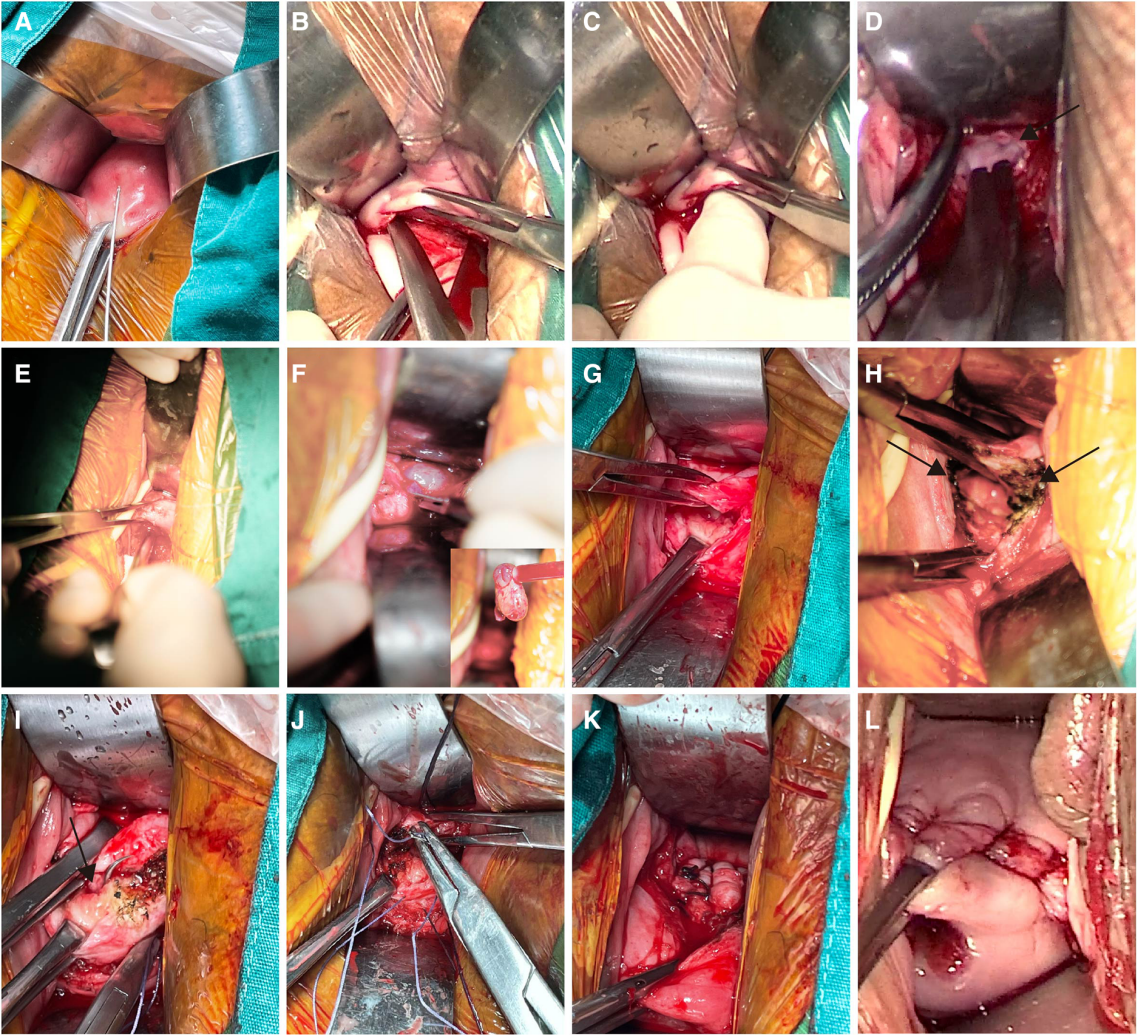
Key points of transvaginal repair. (**A**) Inject 80–100 ml of physiological saline solution containing epinephrine to the vesicocervical space; (**B**) Separate the vesicocervical space sharply; (**C**) Separate the vesicocervical space bluntly; (**D**) Open the peritoneum; (**E**) Perform diagnostic curettage; (**F**) Cut the myometrium of the niche with a polyp in the cavity; (**G**) Remove the weak scar tissue using a knife and dissecting scissors; (**H**) Electro-coagulate the abnormalities in the cavity and cavity extending towards both sides of the corner; (**I**) Identify the cervical ostium; (**J**) Close the resected defect with interrupted full-thickness myometrial sutures; (**K**) All sutures knotted together afterwards; (**L**) Close the peritoneum and vaginal wall.

The procedure for transvaginal repair was as follows: the woman was positioned in lithotomy position, the bladder emptied and 80–100 ml of physiological saline solution containing epinephrine injected to the vesicocervical space (0.3 mg added to 500 ml of saline) to create a separated space (Figure 2A). Subsequently, the cervix was stretched to the upper edge of the cervical vagina; clamped at the fornix; cut the vaginal wall; bluntly and sharply separated the vesicocervical space (Figures 2B,2C), and opened the peritoneum (Figure 2D); performed diagnostic curettage for pathological examination (Figure 2E) and ensure the finger on the anterior wall isthmus could touch the obvious scar-like defect. Nextly, the weak scar tissue was removed using a knife and dissecting scissors (Figures 2F,2G), and sometimes we could see the polyps (Figure 2F), mucus or blood accumulation. All the abnormalities of the niche cavity, such as the stitches, polyps, hyperplasia of the blood vessels, niche cavity extending to both sides were resolved. (Figure 2H). Finally, the cervical ostium was identified(Figures 2I) and the full-thickness myometrial defect was closed with interrupted sutures by using 2–0 absorbable for 4–6 stitches (Figures 2J) with all sutures knotted together afterwards (Figure 2K); the tissues were inverted and aligned with 2–0 absorbable sutures for the peritoneum and vaginal wall (Figure 2L) with an indwelling urinary catheter postoperatively. The weighing method was used to calculate the blood loss amount in the transvaginal group. Intraoperative blood loss amount = total mass of blood gauze-total mass of dry gauze + blood volume in the attractor bottle,, 1 g of blood mass = 1 ml of blood volume, and the total amount of liquid in the attractor bottle minus the total amount of saline and flushing fluid used during operation is the blood volume in the attractor bottle.

**Figure 3:**
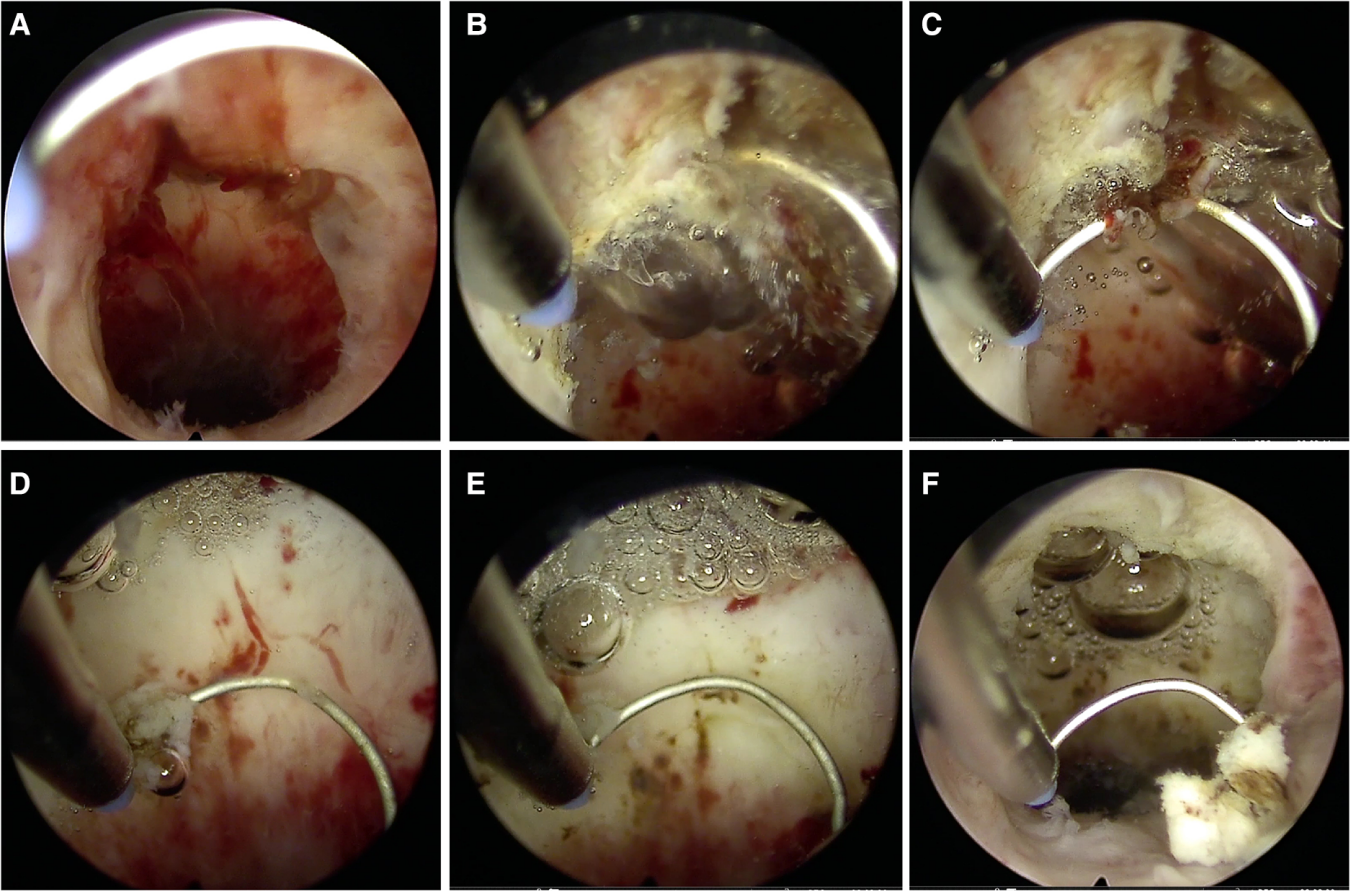
Key points of hysteroscopic resection. (**A**) Niche; (**B,C**) Excision of the niche outflow tract; (**D,E**) Electric burning of the hyperplasia of the blood vessels; (**F**) Niche after the hysteroscopic resection.

**Figure 4:**
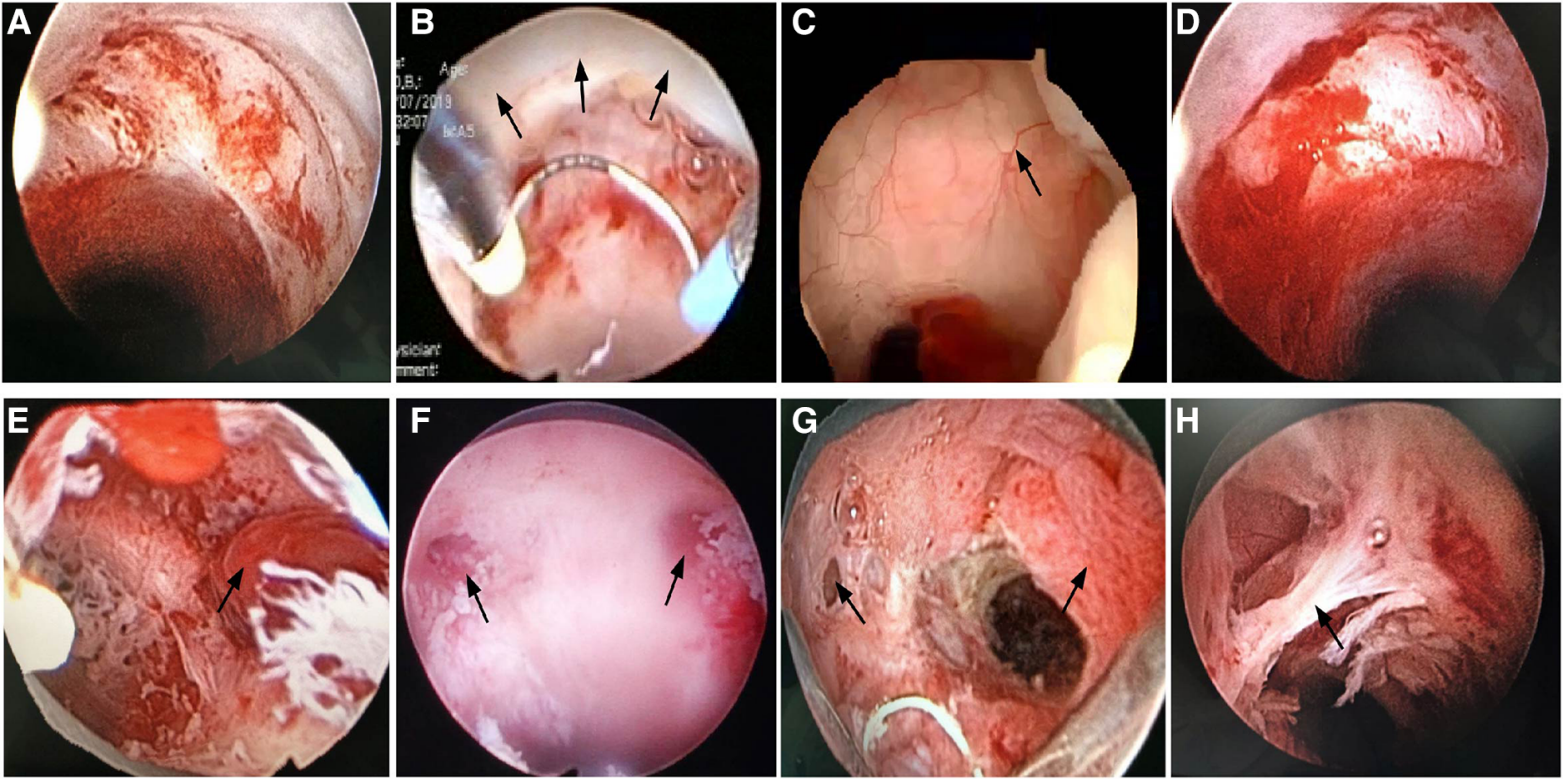
Intraoperative images of hysteroscopic resection. (**A**) Niche; (**B**) Electrocution of the niche outflow tract; (**C**) Abnormally hyperplastic blood vessels in the niche; (**D**) Obvious inflammation in the niche; (**E**) Polyp in the niche; (**F**) Lateral branches on both sides of the niche; (**G**) Polyps in the niche cavity with the right lateral branch; (**H**) Adhesion in the niche cavity.

The procedure for hysteroscopic resection was as follows: the woman was positioned in lithotomy position, the cervix was exposed and expanded using 4–9 Hager's. Subsequently, diagnostic curettage was performed for pathological examination. Next, under the hysteroscope, the niche was seen above the inner opening of the anterior cervix of the uterus and protrudes into the cavity of the serous surface of the uterus (Figure 3A). It may contain dark brown liquid, hyperplastic blood vessels, endometrium, polyps, and other tissues (Figure 4). Bipolar electrocision was performed from the top of the niche directly to the cervical ostium (Figures 3B,3C), with complete excision of the niche outflow tract, and bipolar coagulation was performed for the hyperplasia of the blood vessels (Figures 3D,3E), endometrium, and entire niche cavity (Figure 3F). Owing to the small amount of blood loss in hysteroscopic resection, the visual method was generally adopted. The surgeon roughly estimated the amount of blood loss based on the color of the dilatation fluid during surgery, number of open blood vessels observed *via* endoscopy, and amount of blood accumulated in the vagina.

The postoperative managements were as follows: The transvaginal group received intravenous antibiotics (cefuroxime sodium 3.0 g * 2 days), and the hysteroscopic group received oral antibiotics (levofloxacin 0.4 g * 3 days) to prevent infection. The patients in the transvaginal group, with normal postoperative temperature normal urination regular bowel movement and without complaints of discomfort, were discharged from the hospital. Those in the hysteroscopic group were discharged after being observed for several hours without complaints of discomfort.

### 2.4. Data collection

All data were obtained from the electronic medical record system stored in the database of the sub-specialty clinic. The preoperative baseline data included the patients' age, gravidity, parity, number of CSs, time since last CS, self-reported days of spotting, VAS score for discomfort from spotting, and Likert scale score for menstrual satisfaction. The perioperative data included operation time, intraoperative blood loss, complications, hospitalization duration, and hospitalization costs. The postoperative indicators included self-reported days of spotting, VAS score for discomfort from spotting, and Likert scale score for menstrual satisfaction. In this study, improved spotting symptom was defined as shortening postmenstrual spotting duration byat least 4 days compared with the baseline (30); the improvement rate was calculated as the number of cases with improved postmenstrual spotting symptom over the total number of women at each observation time point. TVUS was performed routinely at 6 months postoperative to assess for the presence of the niche and the postoperative indicators, including the L, W, and D of the niche and RMT, which were compared with the preoperative indicators. When the niche was completely resolved postoperatively, only the RMT was measured. The rate of the resolved niches were calculated as the number of undetectable niche cases on TVUS postoperatively divided by the total number of niche cases confirmed on TVUS preoperatively. The increase value of the postoperative RMT was calculated as the postoperative RMT minus the preoperative RMT.

### 2.5. Outcomes

The primary outcome was postoperative improvement rate of postmenstrual spotting at the 3rd, 6th, 9th, and 12th month after surgery between the two groups. Secondary outcomes were the comparison of pre- and postoperative anatomical indicators, VAS score for discomfort from spotting, Likert scale score for menstrual satisfaction，and perioperative parameters including operation time, intraoperative blood loss, operative complications, hospitalization duration, and hospitalization costs.

### 2.6. Statistical analysis

All statistical tests were conducted using SPSS version 23 (SPSS Inc., Chicago, IL, USA). Normality of the distributions was assessed by inspection of histograms and quantile-quantile plots. Normally distributed continuous variables are presented as mean ± SD and non-normally distributed continuous variables are presented as median (P25, P75). The continuous variables were compared between the groups using the student's t-test or Mann-Whitney U test, as appropriate. Categorical variables were compared using the chi-square test or Fisher's exact test. The generalized estimating equation (GEE) was used to perform statistical analysis on the median of the spotting days repeatedly measured within one year after surgery to obtain a valid standard error. *P*-values of <0.05 were considered statistically significant.

### 2.7. Results

**Figure 5:**
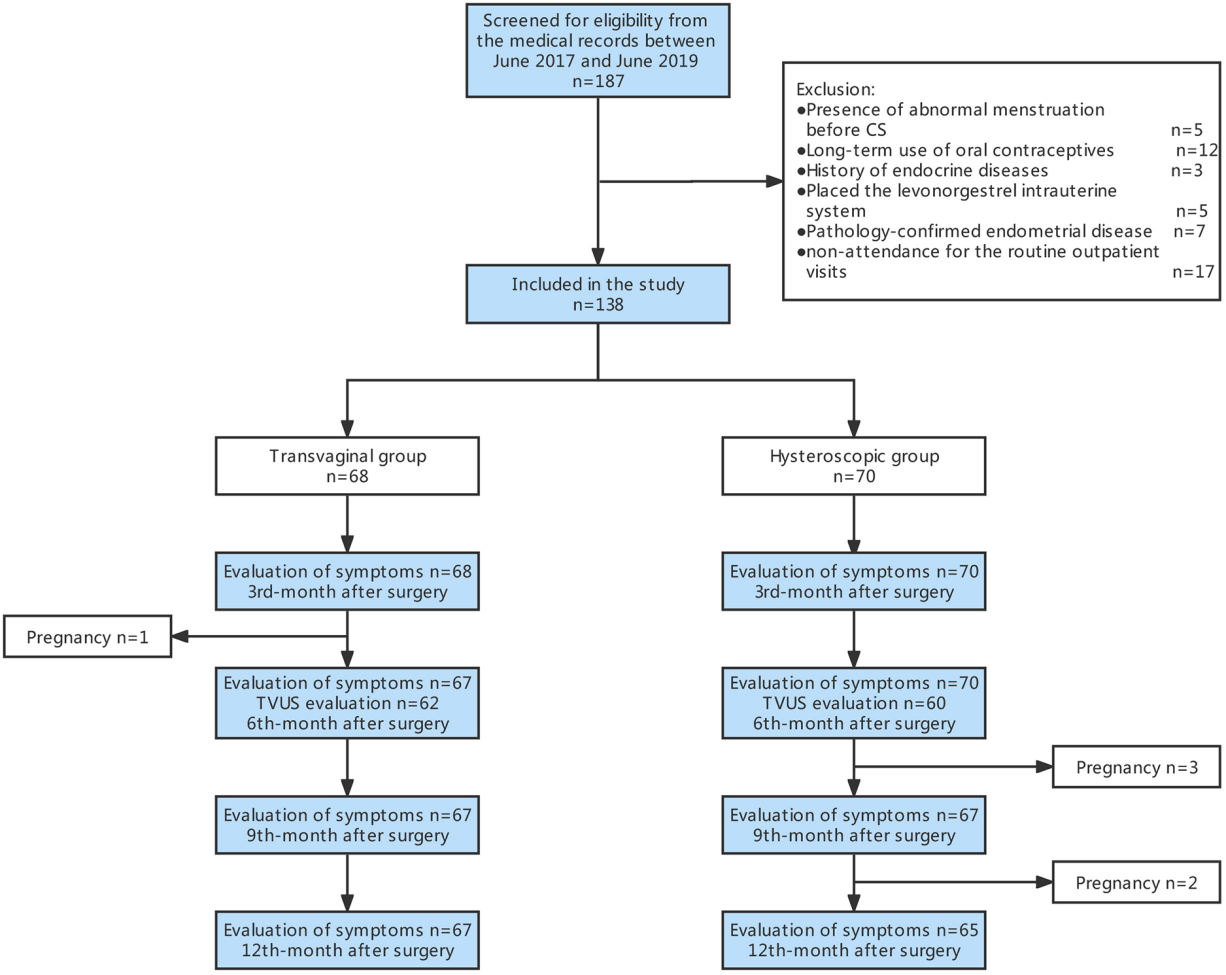
Flow chart.

The flow chart of the study is shown in Figure 5. A total of 187 women underwent transvaginal repair or hysteroscopic resection with RMT at least 2.2 mm from June 2017 to June 2019. Forty-nine patients were excluded based on the exclusion criteria (see Methods). A total of 138 patients were included in this study, including 68 in the transvaginal group and 70 in the hysteroscopic group.

### 2.8. Preoperative baseline characteristics

As shown in Table 1, there were no significant differences between the two groups in terms of age, gravidity, parity, number of CS, the time since last CS, the total days of postmenstrual spotting, the VAS score for discomfort from spotting, the Likert scale score for menstrual satisfaction, and the anatomical indicators of the niches measured on TVUS (all *P* > 0.05).

**Table 1:** Comparison of the preoperative baseline characteristics.

	Transvaginal group	Hysteroscopic group	*P* value^a^
	(*n* = 68)	(*n* = 68)	
Aga (years)	34.1 ± 3.7	34.5 ± 4.5	0.839
Gravidity	2 (1, 3)	2 (1, 3)	0.508
Parity	2 (1, 2)	2 (1, 2)	0.287
Number of cesarean sections	2 (1, 2)	2 (1, 2)	0.424
Time since last caesarean section (years)	6 (3, 9)	5 (3, 9)	0.627
Total days of spotting^b^	8.3 (6.5, 12.4)	8.0 (6.0, 10.0)	0.171
VAS scores of discomfort from spotting (0–10)	6.0 (5.0, 10.0)	6 (4.7, 8.3)	0.813
The Likert scale of menstrual satisfaction (1–5)	2 (1, 2)	2 (1, 2)	0.438
Anatomical indicators of CSD			
Length (mm)	9.6 (7.4, 12.8)	8.2 (7.0, 11.7)	0.142
Width (mm)	14.7 (10.1 18.0)	13.5 (9.0, 16.0)	0.253
Depth (mm)	7.1 (5.7, 8.5)	6.0 (5.0, 7.4)	0.121
RMT (mm)	2.9 (2.6, 3.5)	3.2 (2.7, 3.9)	0.174

Data are reported as means ± standard deviations or as medians (IQRs).

^a^ Student's t-test or Mann-Whitney U test.

^b^ Total days of spotting = sum of the number of spotting days at the end of menstruation and number of intermenstrual spotting days.

VAS, Visual analog scale; TRM, Residual myometrium thickness; IQR, Interquartile range.

### 2.9. Postoperative indicators

#### 2.9.1. Postmenstrual spotting

At the 3rd months after surgery, one woman in the transvaginal group unexpectedly conceived. At the 9th and 12th months after surgery, three and two women in the hysteroscopic group unexpectedly conceived, respectively. Being pregnant, these patients could not complete their postmenstrual spotting symptom report. As shown in Table 2, the improvement rate of postmenstrual spotting at the 3rd, 6th, 9th, and 12th month in the transvaginal group was 87%, 88%, 84%, and 85%, respectively, which was significantly higher than 61%, 68%, 66%, and 68% observed in the hysteroscopic group (*P* < 0.05). At all four observation time points within one year after surgery, the median total of spotting days in the transvaginal group decreased by 8.0, 7.0, 7.0, and 8.0 days compared to baseline, which was significantly higher than 5.0, 5.0, 6.0, and 6.0 days in the hysteroscopic group. The median number of spotting days improved significantly at the 3rd month after surgery and did not change over time in each group (GEE model analysis, *P* > 0.05, Table 3). Additionally, the subjective evaluation indicators (VAS scores of discomforts from spotting and Likert scale of menstrual satisfaction) of the postoperative bleeding symptoms were also better in the transvaginal group than in the hysteroscopic group (Table 2).

**Table 2:** Comparison of the improvement of postmenstrual spotting symptoms.

	Transvaginal group	Hysteroscopic group	*P* value
3rd-month after operation	*n* = 68	*n* = 70	
Improvement rate of spotting symptom^c^	59 (87%)	43 (61%)	0.001^a^
Total days of spotting^d^	0.0 (0.0, 3.0)	3.0 (3.0, 6.0)	0.001^b^
VAS scores of discomforts from spotting (1–10)	0.0 (0.0, 2.0)	2.0 (0.0, 4.0)	<0.001^b^
The Likert scale of menstrual satisfaction (1–5)	5 (4, 5)	4 (3, 5)	0.001^b^
6th-month after operation	*n* = 67	*n* = 70	
Improvement of spotting symptom^c^	59 (88%)	46 (68%)	0.002^a^
Total days of spotting^d^	2.0 (0.0, 3.0)	2.5 (1.0, 6.0)	0.023^b^
VAS scores of discomforts from spotting (1–10)	0.0 (0.0, 2.0)	1.0 (1.0, 3.0)	0.010^b^
The Likert scale of menstrual satisfaction (1–5)	5 (4, 5)	4 (3, 5)	<0.001^b^
9th-month after operation	*n* = 67	*n* = 67	
Improvement of spotting symptom^c^	56 (84%)	44 (66%)	0.017^a^
Total days of spotting^d^	1.0 (1.0, 4.0)	2.0 (0.0, 6.0)	0.013^b^
VAS scores of discomforts from spotting (1–10)	0.0 (0.0, 2.0)	2.0 (0.0, 4.0)	0.020^b^
The Likert scale of menstrual satisfaction (1–5)	5 (4, 5)	4 (4, 4)	0.001^b^
12th-month after operation	*n* = 67	*n* = 65	
Improvement of spotting symptom^c^	57 (85%)	44 (68%)	0.019^a^
Total days of spotting^d^	0.0 (0.0, 3.0)	2.0 (2.0, 5.0)	0.013^b^
VAS scores of discomforts from spotting (1–10)	0.0 (0.0, 2.0)	0.0 (0.0, 4.0)	0.165^b^
The Likert scale of menstrual satisfaction (1–5)	5 (4, 5)	4 (3, 5)	0.016^b^

VAS, Visual analog scale; IQR, Interquartile range.

^a^ Chi-square test.

^b^ Mann-Whitney U test.

^c^ Improvement rate of spotting symptom = the number of cases of the improved postmenstrual spotting symptom divided by the total number of women at each observation time point.

^d^ Total days of spotting = sum of the number of spotting days at the end of menstruation and number of intermenstrual spotting days.

**Table 3:** Analysis of the median of the total days of spotting using the generalized estimating equation (GEE).

Parameters	Valid standard error	95% Wald CI		*P* value
		Upper limit	Lower limit	
Intercept	0.294	3.605	2.453	<0.001
Transvaginal group VS. Hysteroscopic group	0.366	−0.094	−1.528	0.027
The comparison between postoperative follow-up and preoperative				
3rd-month VS. baseline	0.369	6.677	5.229	<0.001
6th-month VS. baseline	0.372	6.676	5.120	<0.001
9th-month VS. baseline	0.387	6.711	5.195	<0.001
12th-month VS. baseline	0.386	6.895	5.383	<0.001
The comparison between different postoperative follow-up times				
6th-month VS. 3rd-month	0.333	0.547	−0.677	0.752
9th-month VS. 6th-month	0.207	0.510	−0.301	0.612
12th-month VS. 9th-month	0.163	0.133	−0.506	0.253

#### 2.9.2. Anatomical indicators

**Figure 6:**
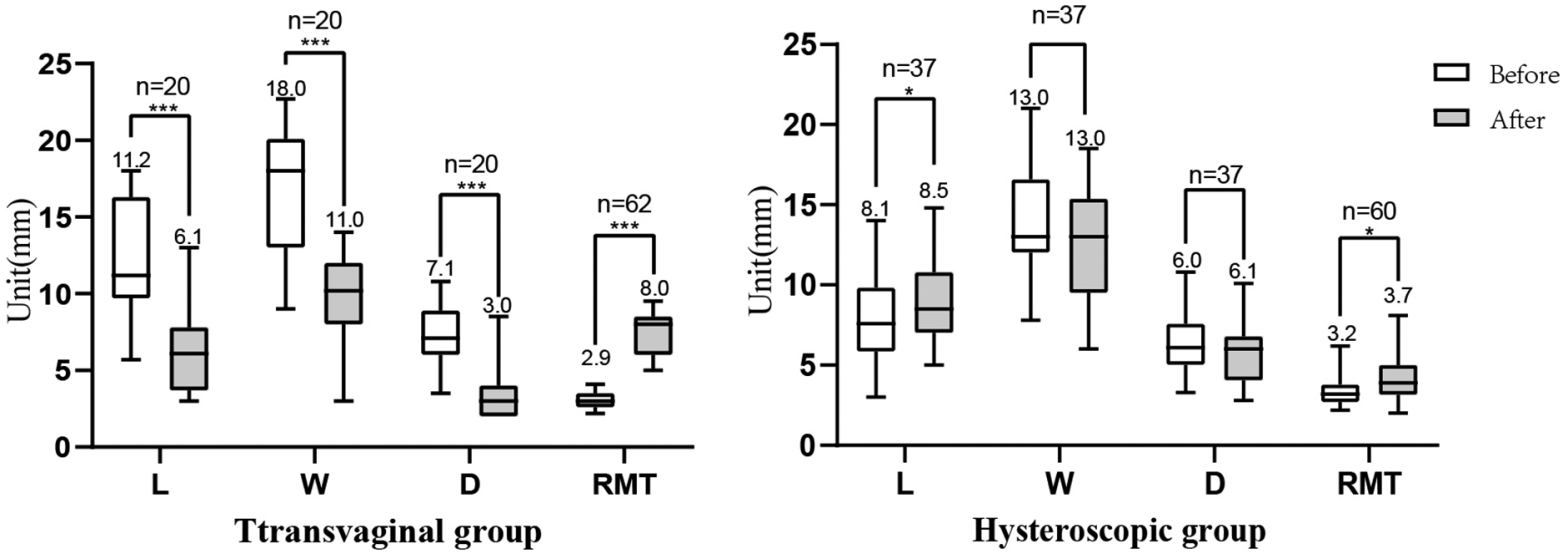
Comparison of the anatomical indicators before and after surgery. (**A**) In the transvaginal group, the median L (11.2, 6.1), W (18.0, 11.0), and D (7.1, 3.0) of the niches significantly decrease compared with the preoperative values, and the median RMT increased from 2.9 mm preoperatively to 8.0 mm postoperatively. (**B**) In the hysteroscopic group, the median L of the niches (8.1, 8.5) significantly increased compared with the preoperative value, and the median RMT increased from 3.2 mm preoperatively to 3.7 mm postoperatively; the median W (13.0,13.0) and D (6.0,6.1) of the niches showed no significant differences. L, length; W, width; D, depth; RMT, residual myometrium thickness. *P < 0.05. ***P < 0.001.

At the 6th month after surgery, six and ten women had no record of TVUS in the transvaginal group and hysteroscopic group, respectively. In the transvaginal group, the median RMT increased from 2.9 mm preoperatively to 8.0 mm postoperatively (*P* < 0.001), In the transvaginal group, 68% of the niche cases (42/62) resolved; the remaining 20 cases still showed niches. The median L, W, and D of these niches significantly decreased compared with the preoperative values (Figure 6A). In the hysteroscopic group, 38% of the niche cases (23/60) resolved; the remaining 37 cases still had niches, with a median RMT increasing from 3.2 mm preoperatively to 3.7 mm postoperatively (*P* < 0.05), the median L of the niches significantly increased compared with the preoperative value (*P* < 0.05); however, the median W and D of the niches were not significantly different before and after surgery (Figure 6B). Comparing across groups after surgery, the resolution rate of the niches and the increase value of the RMT in the transvaginal group were significantly higher than those in the hysteroscopic group (*P* = 0.004 and *P* < 0.001, respectively).

During one year after surgery, a total of six patients (4.35%) has the data about their pregnancies. In the vaginal repair group, one patient achieved pregnancy at the 3th month after surgery with a successful cesarean delivery at 37^+5^ weeks. In the hysteroscopic resection group, five women achieved pregnancies: one at the 12th month experienced a spontaneous miscarriage, one at the 9th month had cesarean delivery at 37 weeks for partial placenta previa and the other three women had successful cesarean deliveries at 37^+4^–38^+3^ weeks.

#### 2.9.3. Perioperative data

All the procedures in the two groups were successfully completed. The following complications occurred in the transvaginal group: fever (*n* = 1), bladder injury (*n* = 1), urinary tract infection (*n* = 1), and vaginal incision infection (*n* = 1); no complications were observed in the hysteroscopic group. Compared to the transvaginal group, the hysteroscopic group had a shorter operative time, less intraoperative blood loss, shorter hospitalization duration, and lower hospitalization costs (*P* < 0.05, Table 4).

**Table 4:** Comparison of the intraoperative information.

	Transvaginal group	Hysteroscopic group	*P* value
	(*n* = 68)	(*n* = 68)	
Operation time (min)	60 (46,70)	21 (15,25)	<0.001^a^
Intraoperative blood loss (ml)	30 (30,50)	10 (5,10)	<0.001^a^
Operative complications	4 (5.9%)	0 (0%)	0.128^b^
Fever	1	0	/
Bladder injury	1	0	/
Urinary tract infection	1	0	/
Vaginal incision infection	1	0	/
Hospitalization duration (days)	6 (4,7)	1 (1,1)	<0.001^a^
Hospitalization costs (RMB)	6,669 (6103,7283)	5,623 (5217,6279)	<0.001^a^

Data are reported as *n* (valid percentages) or as medians (interquartile ranges, IQRs).

^a^ Mann-Whitney U test.

^b^ Fisher's exact test.

## 3. Discussion

According to this retrospective study, both surgical procedures could improve spotting symptom and anatomical structures of the niches, nevertheless, transvaginal repair is superior to hysteroscopic resection. Postoperative spotting days improved significantly at the 3rd month and did not change onwards in each treatment. However, hysteroscopic resection had shorter operation time and hospitalization duration, less operative complications and lower hospitalization costs.

Although both procedures can improve postmenstrual spotting in different manners, transvaginal repair allowed the removal of the niche cavity and surrounding inflammatory and fibrous tissues, correction of the position of uterine flexion, and suturing for the reconstruction of the structure of the lower uterine segment. Therefore, the postoperative bleeding symptom, RMT of the lower uterus, and disappearance rate of the niche, was significantly greater than that with hysteroscopic resection.

As seen on TVUS, transvaginal repair helps restore the anatomical structure of the lower uterine segment. Interestingly, hysteroscopic resection can sometimes improve some values of anatomical indicators. Hysteroscopic surgery does not use suture technology, which theoretically is mostly related to the attenuated lower uterine segment tissue by electrocuting the outflow or inflow tract of the niche, electrocauterizing abnormal tissue in the niche cavity, and destroying the endometrium and fibrous tissue on the entire surface. Therefore, this problem remains controversial among many researchers. Our investigation revealed that the median width of the niches after surgery significantly decreased compared with the preoperative value. The median RMT increased from 3.2 mm before surgery to 3,7 mm after surgery. The possible reason for this phenomenon is the use of electrical equipment to destroy the entire endometrium of the niche cavity surface and fibrous tissue, inducing serious damage to the tissues in the niche cavity, and regeneration of fibrous scar tissue to fill the niche cavity. Previously, Zeller A et al.(31), also showed insignificant improvement in RMT in patients with severe defect (3.7 mm [IQR 2.6–4.8] vs. 2.1 mm [IQR 1.0–3.1], *P* = 0.07). It is important to note that the resolution of symptoms and complete anatomic correction of the defect are not correlated. At 6th-month after hysteroscopic resection, only 38% of the niche cases (23/60) were completely resolved, but symptoms were resolved in 68% (46/70) cases.

To date, there is a lack of supporting evidence for, the postoperative observation interval and observation endpoint of niches remain controversial. In 2016, Zhou et al. (21) followed up 121 patients with niche undergoing vaginal repair and performed TVUS to measure the healing of the anterior wall myometrium of the lower uterine segment at the 1st, 3rd, and 6th months after surgery. They found that healing and RMT of the anterior wall myometrium at the 6th month after surgery were not significantly different from those at the 3rd month after surgery. They also suggested that the incision in the lower uterine segment had been repaired and healed at the 3rd month after surgery, and the anatomical structure had been restored. However, no related research on hysteroscopy has been published. Based on our results, considering the improvement rate of postmenstrual spotting 3 months after surgery reached a stable state in each group, we propose that three months after surgery can be set as a reliable timepoint to assess the symptom improvement indicators in the future.

Our study had a longer observation time after surgery than previous studies, and it not only analyzed the improvement rate of the two procedures in relation to the bleeding symptom but also the effect of time on the improvement indices of the spotting days for the first time. Moreover, subjective evaluation indicators of postmenstrual spotting for the niche were used. The improvement of the anatomical indicators between the two groups before and after surgery was also compared, which was not satisfactorily investigated in previous studies. Several limitations of the present study should also be considered. First, the sample size was relatively small as a result of the retrospective analysis. Second, because most of the patients did not seek conception, we could not assess the outcomes in subsequent pregnancy delivery. Third, the degree of acceptance for the performance of contrast-enhanced sonography in China is low; thus, ultrasonography was performed to measure the corresponding parameters, which might have contributed to a certain rate of missed diagnoses. A consensus statement suggested (32) that recommend delivery by scheduled CS not later than 38 weeks of gestation because of hypothetical increased risk of uterine rupture. However, there are debates about the timing and recommended route of delivery after the managements of niche (33, 34). In the future, further studies must include more samples, and follow more clinical indicators, including the outcome of subsequent pregnancy.

Both surgical procedures could improve spotting symptom and anatomical structures of the niches, but transvaginal repair was superior to hysteroscopic resection. Therefore, during the preoperative conversation on the treatment for women with the niche, physicians and patients can go through the decision-making process together, considering the results of our study (advantages and disadvantages of both surgical approaches) for an informed agreement on treatment choice. Additionally, the 3rd month after surgery can be set as a reliable timepoint for observing to assess symptom improvement indicators of niche. Therefore, we suggest that if the symptoms remain unimproved three months after surgery, additional measures should be considered. Thirdly, the improvement of anatomical indicators of the transvaginal repair are significantly better than those of the hysteroscopic resection. However, the choice of the best surgical procedure is also based on a comprehensive consideration of the woman's future fertility desires.

Both procedures have proven to be effective in reducing niche related postmenstrual spotting duration. Transvaginal repair is superior to hysteroscopic resection, and the postoperative spotting days did not change onwards in each treatment, unlike the treatment of levonorgestrel-releasing intrauterine device (22). For these women with the RMT greater than 2.2 mm, it is important to inform that the shorter operation time and lower costs of the hysteroscopic surgery, along with the patient's fertility desires, so patients and doctors can choose the appropriate operative procedure together.

## 4. Conclusion

Transvaginal repair is better than hysteroscopic resection in improving postmenstrual spotting and the anatomical indicators of the lower uterus. But hysteroscopic resection had the advantages of less operative complications, hospitalization duration and costs. Women with the niche can select the surgical treatment referring to our results for shared decision making with physicians.

## Data Availability

The original contributions presented in the study are included in the article/Supplementary Material, further inquiries can be directed to the corresponding author/s.
